# Combined Effects of Diazoxide and Moderate-Intensity Exercise on the Restoration of Redox Balance Post-Fatigue in Fast- and Slow-Twitch Skeletal Muscles of Hypertensive Rats

**DOI:** 10.3390/biology14111553

**Published:** 2025-11-05

**Authors:** Estefanía Bravo-Sánchez, César J. Nolasco-Ruiz, Sarai Sánchez-Duarte, Mariana Gómez-Barroso, Manuel Alejandro Vargas-Vargas, Christian Cortés-Rojo, Salvador Manzo-Ávalos, Elizabeth Sánchez-Duarte, Alain Raimundo Rodríguez-Orozco, Alfredo Saavedra-Molina, Rocío Montoya-Pérez

**Affiliations:** 1 Instituto de Investigaciones Químico-Biológicas, Universidad Michoacana San Nicolás de Hidalgo, Morelia 58040, Michoacán, Mexico; 1541910b@umich.mx (E.B.-S.); 1209814b@umich.mx (C.J.N.-R.); 1315649c@umich.mx (S.S.-D.); mariana.gomez@ucq.edu.mx (M.G.-B.); manuel.alejandro.vargas@umich.mx (M.A.V.-V.); christian.cortes@umich.mx (C.C.-R.); smanzo@umich.mx (S.M.-Á.); francisco.saavedra@umich.mx (A.S.-M.); 2 Departamento de Ciencias Aplicadas al Trabajo, Universidad Guanajuato Campus León, León 37128, Guanajuato, Mexico; elizabeth.sanchez@ugto.mx; 3 Facultad de Ciencias Médicas y Biológicas “Dr. Ignacio Chávez”, Universidad Michoacana de San Nicolás de Hidalgo, Morelia 58000, Michoacán, Mexico

**Keywords:** antioxidants, oxidants, channels K_ATP_

## Abstract

**Simple Summary:**

Muscle fatigue is linked to redox imbalance and worsens under hypertension due to oxidative stress. This study tested whether diazoxide and moderate exercise could protect skeletal muscles in hypertensive rats after a fatigue protocol. Hypertension increased oxidants and reduced antioxidant defenses. Both interventions, when used alone or in combination, improved antioxidant activity and reduced oxidant levels. The combined treatment showed the most potent effect, with muscle-specific differences: fast-twitch fibers responded better to exercise, while slow-twitch fibers benefited more from the combined approach. These findings demonstrate how this strategy could inform new treatments for hypertension-induced muscle dysfunction.

**Abstract:**

Muscle fatigue, defined as a decline in force generation, is closely linked to redox imbalance—a condition exacerbated by oxidative stress in hypertension. This study investigated the effects of diazoxide administration and moderate-intensity exercise on skeletal muscle redox status following a fatigue protocol in rats with hypertension. Animals were assigned to eight groups: control (CTRL), diazoxide (DZX), exercise (EX), exercise + diazoxide (EX+DZX), hypertension (HTN), hypertension + diazoxide (HTN+DZX), hypertension + exercise (HTN+EX), and hypertension + exercise + diazoxide (HTN+ EX+DZX). Hypertension was induced by a high-salt diet. Diazoxide was administered daily for 14 days, and exercise consisted of moderate treadmill running for 8 weeks. Muscle fatigue was evoked in the extensor digitorum longus (*EDL*) and soleus by repetitive electrical stimulation. Post-fatigue analyses included oxidant levels, catalase activity, and glutathione status. Hypertension increased oxidants and reduced antioxidant defenses in both muscle types. Exercise and diazoxide, alone or in combination, improved redox balance, with the combined treatment providing the most robust protection and exhibiting fiber-specific adaptations. These findings suggest that diazoxide combined with moderate exercise represents a promising therapeutic approach to counteract oxidative stress-related skeletal muscle dysfunction in hypertension.

## 1. Introduction

Muscle fatigue is defined as the progressive decline in the ability of skeletal muscle to generate or maintain force under constant load, a phenomenon resulting from biochemical, metabolic, and redox imbalances [1,2]. Fatigue is a characteristic symptom of pathologies associated with metabolic syndrome, such as obesity, diabetes, and hypertension. It is often accompanied by a reduction in endurance time and an increase in oxidative stress [3,4,5,6].

Skeletal muscles contain distinct fiber types with diverse structural, metabolic, and functional properties [7]. Fast-twitch type II fibers (IIa, IIx, IIB) generate high force but fatigue rapidly, whereas slow-twitch type I fibers contract more slowly and are more fatigue-resistant [2,7,8]. The soleus is mainly composed of type I fibers, while the extensor digitorum longus (EDL) consists predominantly of type II fibers [8,9]. These intrinsic differences influence calcium dynamics and mitochondrial activity, key determinants of redox balance during muscle contraction [2,8,9].

Hypertension reduces muscle strength and fatigue resistance, primarily through mechanisms associated with oxidative stress [10]. This condition involves diminished antioxidant defenses, increased reactive oxygen species (ROS), and consequent impairment of excitation–contraction coupling, calcium homeostasis, and mitochondrial function, all of which accelerate the onset of fatigue [2,10,11].

Exercise has been widely recognized as an effective strategy to improve muscle function in pathological contexts [4]. Adaptations to chronic exercise include angiogenesis, mitochondrial biogenesis, fiber type remodeling, and improved antioxidant defense [12,13,14]. These adaptations are intensity-dependent. Moderate-intensity exercise is considered the most suitable for improving skeletal muscle function, particularly in conditions of hypertension and obesity [10,15]. This type of exercise contributes to increasing muscle strength, improving fatigue resistance, and reducing levels of oxidative stress and inflammation, thus promoting a more balanced muscular and metabolic state compared to low or high intensities [10,15,16].

In addition to exercise, other therapeutic targets have been explored, such as ATP-sensitive potassium channels (K_ATP_), which are widely distributed in various tissues, including skeletal muscle [17]. Channels K_ATP_ play an essential role in the regulation of excitation–contraction coupling and in cellular protection against metabolic stress [17,18]. These channels are located at the sarcolemmal (sarcoK_ATP_) and mitochondrial (mitoK_ATP_) levels, and both have been shown to participate in preserving membrane potential and modulating energy metabolism during conditions of oxidative stress and ischemia [17,18,19]. In cardiac and skeletal muscle, the opening of these channels has been linked to cellular protection mechanisms, maintenance of energy homeostasis, and reduction in fatigue-induced damage [17,18,19,20].

In the use of K_ATP_ channel openers, diazoxide was selected as a pharmacological modulator due to its specific action on mitochondrial K_ATP_ channels, which are crucial for maintaining energy homeostasis and reducing oxidative stress during muscle fatigue [19]. In hypertensive conditions, diazoxide-mediated activation of mitochondrial mitoK_ATP_ has been shown to improve the redox balance and protect against oxidative damage in skeletal muscle [10]. Conversely, in cardiac overload models, mitoK_ATP_ opening by diazoxide reduces mitochondrial ROS and prevents structural damage [21]. Additional evidence links angiotensin II–induced oxidative stress with mitochondrial dysfunction, where mitoK_ATP_ activation contributes to limiting ROS generation [22,23]. Although some clinical observations describe hemodynamic effects such as pulmonary hypertension with diazoxide treatment [24], these findings collectively support its role as a redox modulator through mitoK_ATP_ activation in hypertensive states.

Its administration has shown positive effects on muscle fatigue in healthy animals without altering the antioxidant system [25]. In pathological conditions such as obesity, diabetes, or hypertension, diazoxide not only reduces muscle fatigue but also exerts antioxidant effects in pre-fatigue muscle homogenates [4,5,10]. This makes diazoxide an ideal pharmacological tool for probing the role of K_ATP_ channels in modulating the redox balance during fatigue, complementing the effects of moderate-intensity exercise.

Despite these findings, most studies have assessed the redox status in skeletal muscle before fatigue induction, leaving a critical gap in our understanding of the post-fatigue response in hypertension. Furthermore, the combined effects of exercise and diazoxide on redox homeostasis in different muscle fiber types under post-fatigue conditions remain unexplored. Therefore, this study aimed to evaluate the effects of moderate-intensity exercise and diazoxide on redox balance after a fatigue protocol in fast- and slow-twitch skeletal muscles of hypertensive rats.

## 2. Materials and Methods

### 2.1. Biological Material

Male rats (*Rattus norvegicus*, Wistar strain), approximately 9 weeks old and with an initial average weight of 250 g, were used in this study. The animals were obtained from the University Vivarium Laboratory of the Institute of Neurobiology at the National Autonomous University of Mexico (Juriquilla, Querétaro, México).

The rats were bred conventionally, were not genetically modified, and were free of reported infections. They were maintained under controlled microbiological and parasitological conditions. Upon arrival, the animals were acclimated for two weeks in a 24 °C vivarium, with a 12 h light/dark cycle, a standard rodent diet (Rodent Diet 5001, S.A. de C.V., México City, México), and water available ad libitum.

All procedures were carried out in accordance with the Technical Specifications for the Production, Care, and Handling of Laboratory Animals (NOM-062-ZOO-1999) and were approved by the Institutional Committee for the Use and Care of Laboratory Animals of the Chemical-Biological Research Institute of the Michoacán University of San Nicolás de Hidalgo.

The inclusion criteria were age, sex, strain, and body weight within the specified range. No a priori exclusion criteria were established.

### 2.2. Experimental Assignment and Sample Size

Rats were randomly assigned to eight experimental groups (n = 8 per group at baseline): Control (CTRL), Hypertensive (HTN), Diazoxide (DZX), Exercise (EX), Exercise + diazoxide (EX+DZX), Hypertensive + exercise (HTN+EX), Hypertensive + diazoxide (HTN+DZX), Hypertensive + exercise + diazoxide (HTN+EX+DZX). During the study, three animals per group did not survive to the final blood pressure and metabolic biomarker measurements, primarily due to health reasons or technical complications, resulting in a final sample size of n = 5 rats per group. No data were excluded from statistical analyses.

Cages were rotated periodically within the vivarium, and all procedures were performed on standardized schedules to reduce potential confounding factors.

### 2.3. Sample Size Calculation

The sample size was determined based on our working group’s prior experience with salt-induced hypertension models, in which an average incidence of 80% successful development of hypertension (variable A) and 80% compliance with the training protocol (variable B) has been reported. This empirical data was used to adjust the minimum effective n required per experimental group, considering potential losses during the experiment.

The previously proposed formula [26] was used for the calculation, which has been applied in preclinical studies to estimate the adequate sample size considering expected success rates:

$$X=\frac{N}{\left(\frac{A}{100}\right)\;.(\frac{B}{100})}$$

where

X = final number of animals required per group,

N = minimum number necessary for robust statistical analysis (5),

A = expected rate of hypertension induction (80%),

B = expected rate of compliance with the training protocol (80%).

The calculation resulted in X = 7.81, equivalent to 8 rats per experimental group.

This estimation procedure aligns with the recommendations of the ARRIVE guidelines 2.0 [27] and the principles of NOM-062-ZOO-1999, which suggest using the minimum number of animals necessary to obtain statistically valid results, considering the expected variability of the experimental model.

Given that studies of response to exercise and diazoxide in hypertensive models present moderate biological variability (CV ≈ 15–20%), the adopted sample size guarantees statistical power greater than 80% to detect differences of 25% in the main physiological variables (α = 0.05).

### 2.4. Hypertension Protocol

Hypertension was induced by administering 8% NaCl in the drinking water for 30 days, followed by 4% NaCl for the subsequent eight weeks to maintain the hypertensive condition (J.T. Baker, Phillipsburg, NJ, USA), as previously described protocols [28].

Blood pressure was measured in conscious animals noninvasively (tail cuff) using a CODA single-channel noninvasive blood pressure system (Kent Scientific, Torrington, CT, USA). Rats were placed in a holding rack 5 min before measurements, and systolic and diastolic blood pressure were recorded three times during treatment. All procedures were performed according to the manufacturer’s instructions.

### 2.5. Physical Training Protocol

Exercise sessions were conducted at the beginning of the dark cycle (6:00–8:00 P.M.), a time when rats exhibit physical activity tolerance. The trained groups completed the entire exercise program, while the untrained groups remained inactive.

The moderate-intensity aerobic training program, which lasted 8 weeks, is detailed in Table 1 and followed the previously described protocol [4]. This protocol was developed based on reports of aerobic exercise in rodents, which defined speed and duration ranges compatible with moderate intensity.

All sessions were performed on a Centurfit^®^ treadmill modified with an acrylic chamber divided into eight lanes and equipped with motion sensors that delivered gentle air stimuli when necessary to encourage movement. The treadmill was maintained at a constant inclination of 0° (horizontal) to standardize the mechanical workload among animals and to prevent additional biomechanical stress that could alter fatigue or oxidative stress responses.

The type of training has been associated with improvements in mitochondrial function, increased capillary density, greater fatigue resistance, and reduced oxidative stress and inflammation in skeletal muscle [29,30,31].

### 2.6. Administration of Diazoxide

Diazoxide (D9035, Sigma-Aldrich, St. Louis, MO, USA) was administered intraperitoneally at a dose of 35 mg/kg/day for 14 days, always at 7:00 A.M., to avoid interference with the exercise protocol.

The diazoxide solution was freshly prepared each day by dissolving the compound in dimethyl sulfoxide (DMSO) and diluting it with sterile saline to obtain a final vehicle containing approximately 7% DMSO and 93% saline (*v*/*v*).

It was selected based on experimental precedents in rodents that reported positive effects on muscle function and oxidative stress markers using comparable regimens [4,5]. Furthermore, the literature on pharmacological preconditioning and cellular protection of diazoxide documents the experimental use of doses ranging from 20 to 40 mg/kg i.p., which confer protective effects in various tissue models without significant aqueous toxicity for short to intermediate periods [32,33].

### 2.7. Euthanasia and Tissue Preparation

At the end of the experimental period, rats were euthanized by rapid decapitation. This method was selected to avoid the use of anesthetics that could interfere with the pharmacological effects of diazoxide or alter redox and metabolic parameters in skeletal muscle. Immediately after decapitation, the *EDL* and *soleus* muscles were carefully dissected from both hind limbs.

The isolated muscles were immersed in Krebs–Ringer physiological solution (118 mM NaCl, 4.75 mM KCl, 1.18 mM MgSO_4_, 24.8 mM NaHCO_3_, 1.18 mM KH_2_PO_4_; pH 7.4) and continuously bubbled with carbogen gas (95% O_2_ and 5% CO_2_) to maintain physiological oxygenation and pH levels. Under a stereoscopic microscope, excess connective and adipose tissue was gently removed to preserve muscle fiber integrity, ensuring intact fiber alignment for subsequent fatigue protocol application.

### 2.8. Fatigue Protocol

Skeletal muscle fatigue was induced by repetitive electrical stimulation, following protocols previously reported in the literature [4,5]. Pulses of 100 V and 300 ms duration were applied, with a frequency of 50 Hz for the EDL muscle and 45 Hz for the soleus muscle, using a stimulus isolator unit (Grass Instruments, Quincy, MA, USA).

These parameters were selected to induce sustained and reproducible contractions, allowing the assessment of fatigue resistance in fast (*EDL*) and slow (*soleus*) fibers. Fatigue was defined as a 60–70% decrease from the initial maximum tension, a criterion widely used in isolated skeletal muscle studies to represent a reproducible level of functional decline associated with significant metabolic and ionic disturbances but without causing irreversible structural damage. This range has been consistently applied in experimental fatigue protocols to ensure comparability across studies and to reflect a physiologically meaningful endpoint for biochemical analyses [11,34,35,36,37,38].

After reaching fatigue, the muscles were stored at −80 °C and then homogenized to evaluate biochemical markers, including those related to oxidative stress and antioxidant systems.

### 2.9. Measurement of Oxidants

Oxidant levels were quantified using the intracellular fluorescent probe 2′,7′-dichlorodihydrofluorescein diacetate (H_2_DCFDA; D6883, Sigma Aldrich, St. Louis, MO, USA). 0.5 mg of protein was suspended in 2 mL of a buffer composed of 100 mM KCl, 10 mM HEPES, 3 mM KH_2_PO_4_, and 3 mM MgCl_2_, adjusted to pH 7.4. The sample was incubated with 12.5 μM H_2_DCFDA for 15 min in an ice bath under constant stirring. The fluorescent signal was recorded at 0 and 60 min using a spectrofluorophotometer (Shimadzu RF-5301PC, Kyoto, Japan), with an excitation wavelength of 491 nm and an emission wavelength of 518 nm. The results were expressed as arbitrary units per milligram of protein [39].

### 2.10. Determination of Catalase Activity

Catalase activity was determined by quantifying the conversion of hydrogen peroxide (H_2_O_2_) to molecular oxygen (O_2_) using a Clark-type oxygen electrode coupled to a YSI 5300A biological oxygen monitor (Yellow Springs, OH, USA), following the methodology described above [40] with minor modifications. Briefly, 0.5 mg of protein from muscle homogenate was resuspended in 0.1 M potassium phosphate buffer (pH 7.6) and maintained at 25 °C. The dissolved oxygen signal was monitored for 1 min as a baseline. Subsequently, 6 mM of freshly prepared H_2_O_2_ was added to the reaction chamber, and O_2_ generation was recorded for 2 min. Finally, 1.0 mM sodium azide (S2002, Sigma-Aldrich, St. Louis, MO, USA) was added as an inhibitor. Catalase enzyme activity was calculated using bovine catalase as a reference standard and expressed in arbitrary units per milligram of protein (AU/mg protein).

### 2.11. Determination of Glutathione Status

Total glutathione (GSH + GSSG), oxidized glutathione (GSSG), and reduced glutathione (GSH) levels were quantified using an enzymatic recycling method as previously described [39], with slight modifications. Briefly, 0.5 mg of protein from muscle homogenate was resuspended in a solution containing 0.1% Triton X-100, 0.6% sulfosalicylic acid, and 5 mM disodium EDTA in 50 mM potassium phosphate buffer (pH 7.4). The samples were sonicated using a Branson Sonifier 450 equipped with a tapered microtip (20 W output, continuous duty cycle) for three alternating cycles of sonication and ice incubation. Following this, two freeze–thaw cycles were performed, and the samples were centrifuged at 7200 rpm for 10 min at 4 °C.

Subsequently, 100 μL of the resulting supernatant was mixed with a reaction buffer containing 5 mM disodium EDTA, 0.1 mM 5,5′-dithiobis-(2-nitrobenzoic acid) (DTNB; D8130, Sigma-Aldrich, St. Louis, MO, USA), and 100 mU/mL glutathione reductase in 50 mM potassium phosphate buffer. After 1 min of incubation at room temperature, the reaction was initiated by the addition of 50 μM β-NADPH (N6506, Sigma-Aldrich, St. Louis, MO, USA). Absorbance was monitored at 412 nm over 5 min at 30 °C using a UV-Visible spectrophotometer (Shimadzu UV-2550, Kyoto, Japan).

For GSSG quantification, samples were preincubated with 0.2% 4-vinylpyridine (V3204, Sigma-Aldrich) at room temperature for 1 h to derivatize reduced glutathione. The GSH concentration was obtained by subtracting GSSG from the total glutathione. The redox ratio (GSH/GSSG) was calculated by dividing the concentration of GSH by that of GSSG.

### 2.12. Statistical Analysis

Before applying parametric analyses, the assumptions of normality and homogeneity of variance were checked. Normality was assessed using the Shapiro–Wilk test, and homogeneity using the Brown-Forsythe test. The data generally showed a normal distribution (*p* > 0.05) and homogeneous variances. In cases with slight deviations, the robustness of ANOVA to minor violations of normality was demonstrated. The primary outcomes were compared using factorial analysis of variance (ANOVA) to compare the means of three independent categorical variables (hypertension × exercise × drug). Tukey multiple comparisons were completed when interactions were detected. Statistical significance was set at *p* < 0.05. Data are expressed as mean and standard deviation, with individual data points displayed when possible. All statistical analyses were performed using the software STATISTICA^®^ v 8.0 (Statsoft^®^) and Prism^®^ v.8.0.1 software (GraphPad Inc., La Jolla, CA, USA).

## 3. Results

### 3.1. Effect of Diazoxide and Moderate-Intensity Exercise on Blood Pressure and Weight

To evaluate the systemic effects of the interventions, body weight and blood pressure were monitored throughout the experimental period following the induction of hypertension and subsequent treatments with diazoxide and/or moderate-intensity exercise. These measurements were designed to determine whether the interventions could mitigate hypertensive alterations in physiological parameters. Overall, both diazoxide administration and moderate-intensity exercise attenuated hypertension-induced increases in blood pressure and prevented body weight loss. The combined intervention further improved these parameters, approaching values observed in normotensive controls.

At the end of treatment, the mean weight of the hypertensive group was significantly lower (350.6 ± 28.5 g) compared to the control group (422 ± 37.01 g). However, these values were restored with exercise. Regarding blood pressure, both systolic blood pressure (195.2 ± 12.2 mmHg), diastolic blood pressure (142.4 ± 8.4 mmHg), and mean arterial pressure (160 ± 5.1 mmHg) increased in the hypertensive group. Still, it was slowed by the implementation of the treatments. The results are shown in Table 2.

### 3.2. Effect of Diazoxide and Moderate-Intensity Exercise on Oxidant Levels in Post-Fatigue Skeletal Muscles of Hypertensive Rats

To assess the oxidative stress response after muscle fatigue, oxidant levels were quantified in isolated EDL and soleus muscles obtained from the different experimental groups. This evaluation aimed to determine whether diazoxide and/or moderate-intensity exercise could counteract the pro-oxidant environment induced by hypertension.

Hypertension led to a marked increase in oxidant levels in both fast- and slow-twitch muscles after the fatigue protocol. In contrast, diazoxide and moderate-intensity exercise—either separately or combined—significantly counteracted this effect and restored the redox balance.

**Figure 1:**
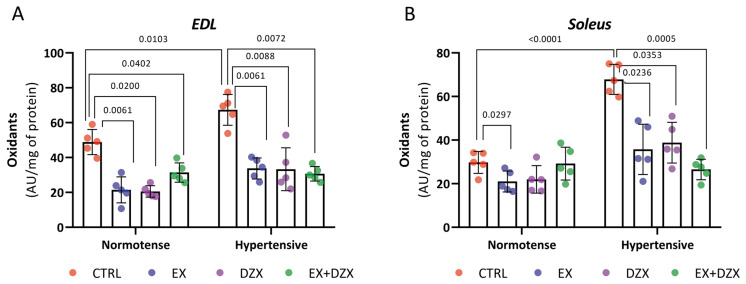
Effect of diazoxide and exercise on oxidant levels in skeletal muscle post-fatigue. This figure shows the levels of oxidants (AU/mg of prot) in the *EDL* muscle post-fatigue (**A**) and the *soleus* muscle post-fatigue (**B**) (AU/mg of prot). CTRL = control; EX = exercise; DZX = diazoxide; EX+DZX= exercise + diazoxide; HTN = hypertension; HTN+EX: hypertension + exercise; HTN+DZX = hypertension + diazoxide; HTN+EX+DZX = hypertension + exercise + diazoxide. The data are expressed as mean  ±  standard deviation (SD); n  =  5. Effects were evaluated using factorial ANOVA. When interactions were detected, Tukey post hoc paired comparisons were completed.

The levels of oxidants in the muscle *EDL* that were subjected to a fatigue protocol are shown in Figure 1A. It is demonstrated that there are higher levels of oxidants in the hypertensive group (*p* = 0.0103) compared to the control group. However, these values are decreased in the hypertensive groups that underwent a moderate-intensity exercise protocol (*p* = 0.0061), diazoxide administration (*p* = 0.0088), and the combination of both (*p* = 0.0072). A similar pattern can be observed in the *soleus* muscle post-fatigue in Figure 1B. Oxidant levels were elevated in the hypertensive group compared to the control group (*p* < 0.0001). However, these levels were reduced in the hypertensive + diazoxide group (*p* = 0.0353), hypertensive + exercise group (*p* = 0.0236), and hypertensive + exercise + diazoxide group (*p* = 0.0005). These results demonstrate that oxidants increased in the hypertensive groups after a fatigue induction protocol in both fast-twitch and slow-twitch muscles, and the treatments of exercise and diazoxide reversed this.

### 3.3. Effect of Diazoxide and Moderate-Intensity Exercise on Catalase Activity in Post-Fatigue Skeletal Muscles of Hypertensive Rats

To explore the antioxidant enzymatic response to hypertension and treatment, catalase activity was evaluated in post-fatigue EDL and soleus muscles. A decline in catalase activity was observed in hypertensive rats following the fatigue protocol, indicating impaired antioxidant defense in skeletal muscle. Both diazoxide treatment and moderate-intensity exercise enhanced catalase activity, and their combination produced the most pronounced restoration in enzymatic function.

**Figure 2:**
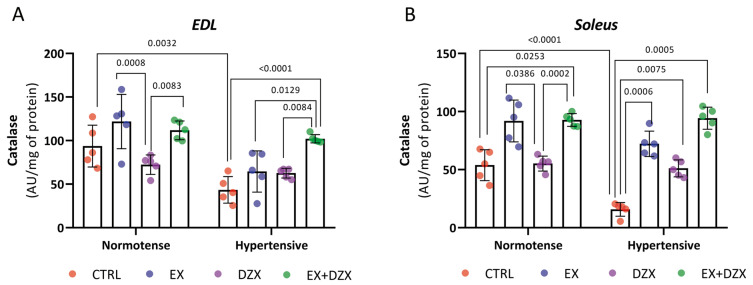
Effect of diazoxide and exercise on catalase activity in skeletal muscle post-fatigue. This figure illustrates the activity of the catalase enzyme (AU/mg of prot) in the *EDL* muscle post-fatigue (**A**) and the *soleus* muscle post-fatigue (**B**) (AU/mg of prot). CTRL = control; EX = exercise; DZX = diazoxide; EX+DZX= exercise + diazoxide; HTN = hypertension; HTN+EX: hypertension + exercise; HTN+DZX = hypertension + diazoxide; HTN+EX+DZX = hypertension + exercise + diazoxide. The data are expressed as mean  ±  standard deviation (SD); n  =  5. Effects were evaluated using factorial ANOVA. When interactions were detected, Tukey post hoc paired comparisons were completed.

The results of the catalase activity of the *EDL* muscle post-fatigue are shown in Figure 2A. As can be observed, the hypertensive group (*p* = 0.0032) has decreased activity compared to the control group, indicating a reduction in the antioxidant system of catalase. On the contrary, in the hypertensive groups treated with exercise and diazoxide (*p* < 0.0001), an increase in catalase activity was observed, demonstrating that the treatments are beneficial in restoring the enzyme’s activity. In Figure 2B, the results for the *soleus* muscle post-fatigue are shown. Catalase activity was significantly decreased in the hypertensive group (*p* < 0.0001) versus the control group. For the hypertensive + exercise (*p* = 0.0006), hypertensive + diazoxide (*p* = 0.0075), and hypertensive + exercise + diazoxide (*p* = 0.0005) groups, catalase activity increased significantly.

### 3.4. Effect of Diazoxide and Moderate-Intensity Exercise on Glutathione Status in Post-Fatigue Skeletal Muscles of Hypertensive Rats

To further characterize alterations in redox balance, total, reduced (GSH), and oxidized (GSSG) glutathione levels were measured in post-fatigue EDL and soleus muscles from the different experimental groups. This approach aimed to determine whether diazoxide and moderate-intensity exercise could restore the glutathione-dependent antioxidant system, which is affected by hypertension.

Hypertension disrupted glutathione homeostasis in both fast- and slow-twitch muscles, as reflected by decreased total glutathione levels, reduced glutathione levels, elevated oxidized glutathione, and a diminished GSH/GSSG ratio. Treatments with diazoxide and moderate-intensity exercise—particularly in combination—partially restored this redox balance.

**Figure 3:**
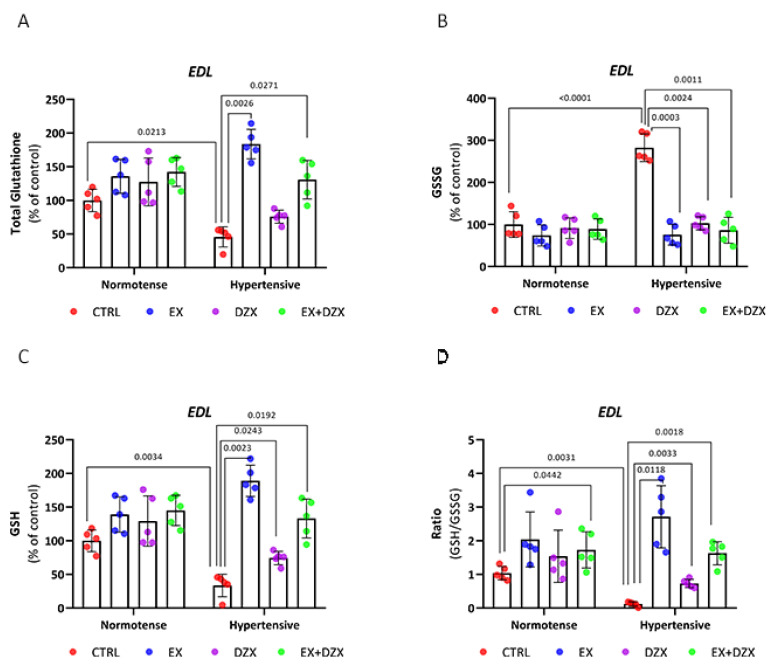

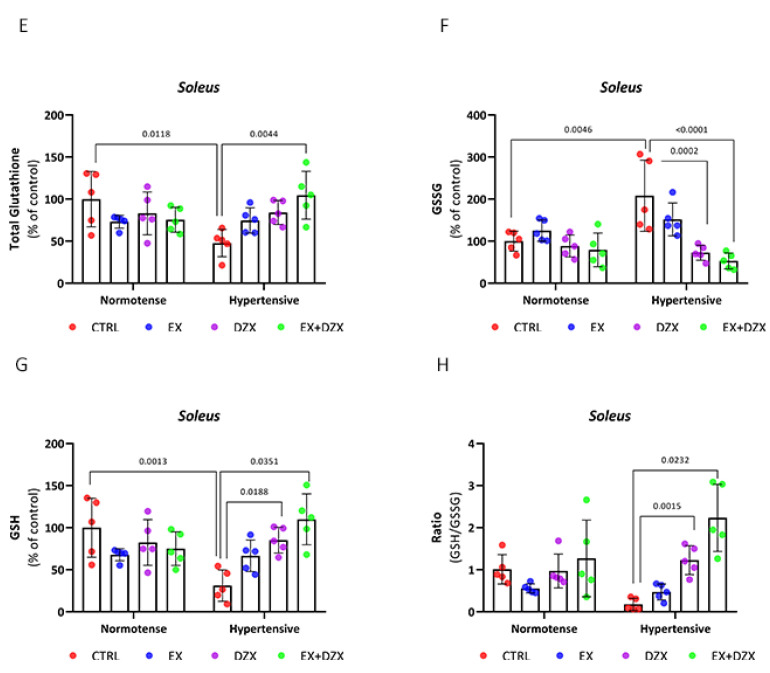
Effect of diazoxide and exercise on glutathione status in skeletal muscle post-fatigue. This figure shows the levels of total glutathione in the *EDL* muscle (**A**), oxidized glutathione in the *EDL* muscle (**B**), reduced glutathione in the *EDL* muscle (**C**), GSH/GSSG ratio in the *EDL* muscle (**D**), glutathione total in the *soleus* muscle (**E**); oxidized glutathione in the *soleus* muscle (**F**); reduced glutathione in *soleus* muscle (**G**); and GSH/GSSG ratio in the *soleus* muscle (**H**). CTRL = control; EX = exercise; DZX = diazoxide; EX+DZX= exercise + diazoxide; HTN = hypertension; HTN+EX: hypertension + exercise; HTN+DZX = hypertension + diazoxide; HTN+EX+DZX = hypertension + exercise + diazoxide. The data are expressed as mean  ±  standard deviation (SD); n  =  5. Effects were evaluated using factorial ANOVA. When interactions were detected, Tukey post hoc paired comparisons were completed.

In Figure 3A, the results of total glutathione for the post-fatigue *EDL* muscle are shown, where a decrease is observed in the hypertensive group compared to the control group (*p* = 0.0213). However, this is improved in the hypertensive + exercise (*p* = 0.0026) and hypertensive + exercised + diazoxide (*p* = 0.0271) groups. In oxidized glutathione (Figure 3B), the hypertensive group exhibits higher levels compared to the control group (*p* < 0.0001). With the administration of diazoxide (*p* = 0.0024), the implementation of exercise (*p* = 0.0033), and the combination of both (*p* = 0.0011), the level of significance is significantly decreased. In contrast, reduced glutathione (Figure 3C) decreased in the hypertensive group (*p* = 0.0034); however, with exercise (*p* = 0.0023), with diazoxide (*p* = 0.0243), and the combination of diazoxide + exercise (*p* = 0.0192), GSH values increased significantly. In the GSH/GSSG ratio (Figure 3D), the hypertensive group (*p* = 0.0034) showed decreased values considerably; however, moderate-intensity exercise (*p* = 0.0118), diazoxide (*p* = 0.0033), and the combination of exercise + diazoxide (*p* = 0.0018) increased the activity of the glutathione antioxidant system, indicating that the treatments had beneficial effects on the EDL muscle post-fatigue during hypertension.

Total glutathione results for the post-fatigue *soleus* muscle are shown in Figure 3E. In the hypertensive group (*p* = 0.0118), total glutathione values decreased dramatically compared to the control group. However, statistically significant differences were observed in the hypertensive + exercise + diazoxide group (*p* = 0.0044). In oxidized glutathione (Figure 3F), the hypertensive group (*p* = 0.0046) showed elevated levels compared to the control. However, with the administration of diazoxide (*p* = 0.0002) and the combination of exercise + diazoxide (*p* < 0.0001), the GSSG levels decreased significantly. In contrast, in reduced glutathione (Figure 3G), GSH values decreased in the hypertensive group compared to the control group (*p* = 0.0013). However, in the hypertensive + diazoxide (*p* = 0.0188) and hypertensive + exercise + diazoxide (*p* = 0.0351) groups, GSH values increased significantly. Similar behavior was observed in the GSH/GSSG ratio in the hypertensive group, with decreased values. In this case, the increase in the ratio was only observed in the hypertensive + exercise + diazoxide group (*p* = 0.0232) and not with the individual treatments.

With the experimental results available for the redox state of glutathione, we can determine that both in the *EDL* and *soleus* muscle post-fatigue, during hypertension, the activity of this antioxidant decreases; however, with the implementation of moderate-intensity exercise and the administration of diazoxide, mainly together, the activity of the glutathione antioxidant system improves during hypertension, after a muscle fatigue induction protocol.

## 4. Discussion

Hypertension exacerbates skeletal muscle fatigue by increasing oxidant production and impairing antioxidant defenses, affecting both fast- and slow-twitch muscles. ROS are continuously produced in skeletal muscle at rest and during contractile activity, where they play a dual role: regulating blood flow, force production, and exercise-induced adaptations [13]. However, excessive ROS generation in pathological conditions such as hypertension shifts this balance toward oxidative stress.

Our group was the first to report the beneficial effects of moderate-intensity exercise and the pharmacological opening of K^+^ channels on impaired muscle function in sodium-induced hypertension, considering the context of muscle redox status [10]. The present study expands on this evidence by characterizing post-fatigue responses in skeletal muscle, showing that hypertension markedly increases oxidant levels in both the EDL and soleus muscles [Figure 1], while diazoxide and/or moderate-intensity exercise effectively restore redox homeostasis.

This redox imbalance has direct functional consequences, as ROS are closely linked to contractile impairment. A dose-dependent relationship has been described between ROS production, decreased muscle strength, and reduced fatigue resistance [41]. Similarly, in obese rats, interventions with diazoxide, exercise, or their combination reduced ROS and lipid peroxidation in oxidative and glycolytic muscles, before and after fatigue [4]. These findings reinforce the systemic vulnerability of skeletal muscle to oxidative stress and redox alterations in metabolic disorders.

The protective effects observed here can be partly explained by K^+^ channel physiology. The opening of these channels is critical under metabolic stress (e.g., ischemia, fatigue), as it preserves ATP levels and prevents irreversible damage [42,43,44]. Furthermore, K^+^ channels regulate glucose uptake and K+ flux in muscle fibers [45]. Notably, diazoxide reduced ROS production in the skeletal muscle fibers of mice by activating mitoK_ATP_ channels [25]. Consistent with these mechanisms, pre- and post-fatigue analysis (Table S1) confirmed that hypertensive muscle accumulates a greater oxidative load than controls, supporting previous reports that repetitive stimulation induces hypoxia, ROS accumulation, and tissue injury [20,46,47]. In contrast, the combination of diazoxide and exercise not only prevented the development of ROS but also, in late life, reduced oxidant levels below baseline, suggesting a stress-preconditioning-like effect. This concept aligns with hormesis, according to which moderate stress, such as exercise-induced ROS, triggers protective adaptations through cellular signaling [13,48,49].

Among enzymatic defenses, catalase activity was profoundly altered by hypertension. As shown in Figure 2, catalase, a key enzyme in H_2_O_2_ detoxification [50,51], was significantly reduced in hypertensive rats, whereas both exercise and diazoxide restored its activity, especially when combined. These results are consistent with evidence that exogenous superoxide dismutase (SOD) reduces blood pressure in hypertensive models [52,53]. However, they also highlight that an isolated increase in superoxide dismutase, without compensatory catalase activity, can lead to excess H_2_O_2_ [54,55]. Catalase recovery showed fiber-specific patterns, being most pronounced in the soleus muscle with diazoxide and in the EDL muscle with exercise, indicating that slow-twitch oxidative fibers benefit more from mitochondrial modulation [56,57,58,59,60].

The glutathione system, a key non-enzymatic antioxidant, also showed marked modulation. Hypertension decreased GSH and the GSH/GSSG ratio in both muscle types [Figure 3], consistent with previous studies associating glutathione imbalance with pathological remodeling [61]. In the EDL muscle, exercise effectively increased GSH and restored the GSH/GSSG ratio, whereas in the soleus, glutathione balance improved only when exercise and diazoxide were combined. This highlights fiber-specific antioxidant regulation, where glycolytic fibers rely more on cytosolic pathways and oxidative fibers on mitochondrial buffering [61,62,63]. Furthermore, S-glutathionylation, a GSH-dependent reversible modification, is readily produced in fast-twitch fibers during exercise, increasing Ca^2+^ sensitivity and contractile force [64,65].

Notably, the differential recovery of catalase and glutathione highlights their complementary yet distinct regulatory roles. Catalase responds rapidly to stress-modifying interventions, while glutathione reflects longer-term, fiber-specific redox adjustments. Taken together, our findings support a model in which exercise and diazoxide provide synergistic protection against hypertension-induced oxidative stress and fatigue by converging on complementary pathways: cytosolic (*Nrf2*-mediated) and mitochondrial (mitoK_ATP_-dependent). Fiber-specific responses underscore the importance of considering muscle phenotype when designing therapeutic strategies [66,67].

Taken together, our findings support a model in which exercise and diazoxide provide synergistic protection against hypertension-induced oxidative stress and fatigue by converging on complementary pathways: cytosolic (*Nrf2*-mediated) and mitochondrial (mitoK_ATP_-dependent). Fiber-specific responses underscore the importance of muscle phenotype: fast-twitch fibers, which are more vulnerable to ROS accumulation, primarily benefit from exercise-induced cytosolic adaptations, whereas slow-twitch fibers require mitochondria-targeted interventions, such as diazoxide, to restore redox balance.

Although our findings in the animal model (35 mg/kg of diazoxide) are promising, translation to human clinical use requires caution. In humans, diazoxide has been used in hypertensive crises at doses of 5 mg/kg via infusion [68] and in oral administration for other indications at recommended doses of ~5–10 mg/kg/day [69,70,71]. Therefore, species differences in pharmacokinetics, metabolism, and K^+^-channel expression imply that direct extrapolation of our 35 mg/kg animal dose to humans with hypertension-induced muscle fatigue should be approached with caution. Future studies should determine safe and effective human doses, pharmacodynamics, and long-term safety in this context.

Translationally, the findings suggest that combining pharmacological and lifestyle strategies may be an effective approach to protecting skeletal muscle in hypertension. Exercise prescription remains a cornerstone in the management of hypertension. Post-fatigue results indicate that diazoxide can modulate oxidant production in hypertensive muscle, suggesting an additive effect when combined with exercise. However, our study did not directly assess Nrf2 activation, mitochondrial function, or calcium regulation, so any inferences regarding these mechanisms should be considered exploratory. Future studies should investigate these mechanistic pathways and assess causality to confirm the observed effects and refine therapeutic applications.

## 5. Conclusions

This study demonstrates that both diazoxide and moderate-intensity exercise independently improve the redox balance of skeletal muscle after fatigue in hypertensive rats, with a more pronounced effect when applied in combination. The observed antioxidant enhancement was fiber-type dependent, being more robust in fast-twitch (*EDL*) than in slow-twitch (*soleus*) muscle. The interventions reduced oxidant levels and restored the activity of key antioxidant systems, including catalase and glutathione, following fatigue-induced oxidative stress.

These findings suggest a synergistic protective effect of moderate exercise and pharmacological activation of the mitoK_ATP_ channel, which may serve as a basis for therapeutic strategies aimed at preserving skeletal muscle function in hypertension and related metabolic disorders. The differential response between muscle fiber types further emphasizes the need for personalized approaches in redox-based interventions.

## Figures and Tables

**Table 1:** Moderate intensity exercise protocol.

Week (5 d/wk)	Speed Per Day (m/min)	Time Per Day (min)
Acclimatization	10	5
1	10	10
2	10	15
3	10	15
	17	5
4	10	15
	17	10
5	10	15
	17	10
	22	5
6	10	20
	17	15
	22	5
7	10	20
	17	15
	22	5
8	10	20
	17	15
	22	5

**Table 2:** Body weight and blood pressure.

Groups	Weight (g)	SBP (mmHg)	DBP (mmHg)	MAP (mmHg)
CTRL	422 ± 37.01	126.2 ± 3.1	88.8 ± 6.3	101.2 ± 3.9
EX	458.6 ± 19.3	125.4 ± 3.5	88.4 ± 12.1	100.7 ± 8.8
DZX	456 ± 15.1	112.4 ± 8.2	72.6 ± 12.6	85.8 ± 10.0
EX+DZX	456.4 ± 26.1	117.4 ± 6.4	78.2 ± 13.04	91.2 ± 9.8
HTN	350.6 ± 28.5 *	195.2 ± 12.1 *	142.4 ± 8.4 *	160 ± 5.1 *
HTN+EX	407.8 ± 16.7 ^&^	171.2 ± 7.8 *^,&^	119.4 ± 7.6 *	136.6 ± 6.3 *^,&^
HTN+DZX	367.2 ± 11.38 *	145.2 ± 13.3 *^,&^	114.2 ± 9.9 *	124.5 ± 10.5 *^,&^
HTN+EX+DZX	407.8 ± 16.7 ^&^	147.6 ± 12.7 *^,&^	118 ± 11.4 *	127.8 ± 4.5 *^,&^

Effect of diazoxide and exercise on weight, systolic blood pressure (SBP), diastolic blood pressure (DBP), and mean arterial pressure (MAP). CTRL = control; EX = exercise; DZX = diazoxide; EX+DZX= exercise + diazoxide; HTN = hypertension; HTN+EX: hypertension + exercise; HTN+DZX = hypertension + diazoxide; HTN+EX+DZX = hypertension + exercise + diazoxide. The data are expressed as a mean ± standard deviation (SD); n  =  5. Effects were evaluated using factorial ANOVA. When interactions were detected, Bonferroni’s multiple comparisons test was performed and reported, such that * *p* ≤ 0.05 vs. CTRL ^&^ *p* ≤ 0.05 vs. HTN.

## Data Availability

All data and materials used in the analysis are available in some form to any researcher for purposes of reproducing or extending the analysis.

## Supplementary Materials

The following supporting information can be downloaded at: https://www.mdpi.com/article/10.3390/biology14111553/s1, Table S1: Oxidant levels in the muscles EDL and soleus before fatigue and post fatigue; Table S2: Catalase activity in the muscles EDL and soleus before fatigue and post fatigue; Table S3: Glutathione redox status in the muscles EDL and soleus before fatigue and post fatigue.

## Abbreviations

The following abbreviations are used in this manuscript:

EDL	Extensor digitorum longus
ROS	Reactive oxygen species
K_ATP_	ATP-sensitive potassium
Sarco-K_ATP_	Sarcolemmal ATP-sensitive potassium
Mito-K_ATP_	Mitochondrial ATP-sensitive potassium
GSSG	Oxidized glutathione
GSH	Reduced glutathione
H_2_O_2_	Hydrogen peroxide
O_2_	Molecular oxygen
SOD	Superoxide dismutase
mPTP	Mitochondrial permeability transition pore
