# Supplementary material for: Combined Effects of Diazoxide and Moderate-Intensity Exercise on the Restoration of Redox Balance Post-Fatigue in Fast- and Slow-Twitch Skeletal Muscles of Hypertensive Rats
Source: Biology (Basel). 2025 Nov 5;14(11):1553. doi: 10.3390/biology14111553 (PMC12650644; doi:10.3390/biology14111553)
Supplement: Supplementary file 1 [file biology-14-01553-s001.zip › biology-3888979-supplementary.pdf]

Supplementary material

Original research article

Combined Effects of Diazoxide and Moderate-Intensity Exercise on the Restoration of Redox Balance Post-Fatigue in Fast- and Slow-Twitch Skeletal Muscles of Hypertensive Rats

Supplementary tables

Table S1. Oxidant levels in the muscles *EDL* and *soleus* before fatigue and post-fatigue. CTRL: control; EX: exercise; DZX: diazoxide; EX+DZX: exercise + diazoxide; HTN: hypertension; HTN+EX: hypertension + exercise; HTN+DZX: hypertension + diazoxide; HTN+EX+DZX: hypertension + exercise + diazoxide.

Groups	*EDL*		*Soleus*	
	Before fatigue	Post fatigue	Before fatigue	Post fatigue
**CTRL**	20.4 ± 4.06	48.8 ± 7.1*	12.8 ± 1.7	29.7 ± 5.03*
**EX**	22.54 ± 5.8	21.4 ± 7.4	18.9 ± 2.9	21.03 ± 4.8
**DZX**	13.9 ± 3.5	20.5 ± 3.3	12.8 ± 3.9	21.9 ± 6.2
**EX+DZX**	22.4 ± 4.6	31.4 ± 5.5	17.8 ± 4.1	29.2 ± 7.5
**HTN**	48.9 ± 6.9	67.3 ± 8.8*	43.2 ± 5.9	67.8 ± 6.8 *
**HTN+EX**	41.5 ± 6.2	33.7 ± 6.005	36.1 ± 5.02	35.7 ± 11.5
**HTN+DZX**	36.6 ± 3.4	33.3 ± 12.2	30.08 ± 3.1	38.8 ± 9.3
**HTN+EX+DZX**	21.4 ± 2.2	30.7 ± 4.1	19.1 ± 5.3	26.5 ± 4.7

The data are expressed as a mean ± standard deviation (SD); $n = 5$. Effects were evaluated using factorial ANOVA. When interactions were detected, Tukey post hoc paired comparisons were completed and reported such that *$p \leq 0.05$ before fatigue versus post-fatigue.

Table S2. Catalase activity in the muscles *EDL* and *soleus* before fatigue and post-fatigue. CTRL: control; EX: exercise; DZX: diazoxide; EX+DZX: exercise + diazoxide; HTN: hypertension; HTN+EX: hypertension + exercise; HTN+DZX: hypertension + diazoxide; HTN+EX+DZX: hypertension + exercise + diazoxide.

Groups	*EDL*		*Soleus*	
	Before fatigue	Post fatigue	Before fatigue	Post fatigue
**CTRL**	42.03 ± 3.3	93.8 ± 23.9	25.9 ± 6.7	53.8 ± 13.3*
**EX**	86.7 ± 9.2	121.9 ± 31.1	38.3 ± 7.9	91.9 ± 18.02*
**DZX**	52.7 ± 72.4	72.4 ± 11.09*	39.2 ± 5.1	55.2 ± 6.4
**EX+DZX**	76.9 ± 15.2	111.9 ± 10.7*	61.7 ± 11.08	92.8 ± 5.5*
**HTN**	13.5 ± 4.5	43.4 ± 15.1	4.2 ± 3.2	15.7 ± 5.8
**HTN+EX**	26.9 ± 9.1	64.5 ± 23.6	21.02 ± 5.9	72.2 ± 10.9*
**HTN+DZX**	46.1 ± 9.7	62.6 ± 5.5*	28.3 ± 8.1	51.1 ± 7.2*
**HTN+EX+DZX**	95.4 ± 18.7	102.1 ± 4.7	61.7 ± 12.07	94.3 ±9.5

The data are expressed as a mean ± standard deviation (SD); $n = 5$. Effects were evaluated using factorial ANOVA. When interactions were detected, Tukey post hoc paired comparisons were completed and reported such that *$p \leq 0.05$ before fatigue versus post-fatigue.

Table S3. Glutathione redox status in the muscles *EDL* and *soleus* before fatigue and post-fatigue. CTRL: control; EX: exercise; DZX: diazoxide; EX+DZX: exercise + diazoxide; HTN: hypertension; HTN+EX: hypertension + exercise; HTN+DZX: hypertension + diazoxide; HTN+EX+DZX: hypertension + exercise + diazoxide.

Groups	*EDL*		*Soleus*	
	Before fatigue	Post fatigue	Before fatigue	Post fatigue
**Glutathione total (% of control)**				
**CTRL**	100 ± 9.8	100 ± 16.6	106 ± 8.4	100 ± 32.9
**EX**	112.2 ± 17.2	135.9 ± 25.2	118.8 ± 15.2	73.2 ± 7.7
**DZX**	93.8 ± 14.1	127.5 ±35.5	111.9 ± 11.2	83.05 ± 25.4
**EX+DZX**	129.2 ± 14.7	142.3 ± 21.3	115.4 ± 15.9	75.4 ± 14.8*
**HTN**	70.33 ± 10.5	45.7 ± 14.9	81.6 ± 9.6	47.8 ± 16.3*
**HTN+EX**	88.5 ± 12.003	183.6 ± 21.9*	90.4 ± 11.9	74.6 ± 15.02
**HTN+DZX**	95.08 ± 10.7	75.84 ± 9.7	99.8 ± 8.1	84.09 ± 14.2
**HTN+EX+DZX**	108.2 ± 12.2	130.8 ± 28.5	113.8 ± 10.4	104.6 ± 28.4
**Glutathione oxidized (GSSG) (% of control)**				
**CTRL**	100 ± 6.5	100 ± 30.6	98 ± 10.9	100 ± 23.6
**EX**	110.9 ± 5.9	74.2 ± 25.07	116.6 ± 10.3	125.2 ± 25.3
**DZX**	94.8 ± 17.03	91.3 ± 24.4	95.7 ± 20.8	88.6 ± 26.1
**EX+DZX**	118.2 ± 7.7	89.1 ± 24.1	76.7 ± 10.1	79.3 ± 40.009
**HTN**	167.8 ± 7.6	282.6 ± 32.9*	128.9 ± 12.5	208.2 ± 84.6
**HTN+EX**	110.7 ± 12.1	75.9 ± 24.7	75.06 ± 11.3	151.8 ± 38.9
**HTN+DZX**	130.3 ± 9.8	103.01 ± 15.7	59.6 ± 7.8	72.75 ± 17.9
**HTN+EX+DZX**	54.4 ± 10.2	86.3 ± 30.8	57.8 ± 5.08	53.08 ± 18.9
**Glutathione reduced (GSH) (% of control)**				
**CTRL**	92.4 ± 7.2	100 ± 16.1	106 ± 8.06	100 ± 35.1
**EX**	118.4 ± 10.1	139.08 ± 26.2	179.9 ± 20.6	67.8 ± 7.1*
**DZX**	99.4 ± 11.4	129.3 ± 37.4	163.9 ± 26.9	82.4 ± 27.2
**EX+DZX**	132.5 ± 9.4	145.06 ± 22.5	206.6 ± 31.9	75.07 ± 19.9*
**HTN**	11.4 ± 3.3	33.6 ± 16.6	21.7 ± 5.4	31.1 ± 18.6
**HTN+EX**	73.8 ± 8.5	189.1 ± 23.2*	103.7 ± 21.4	66.6 ± 18.7
**HTN+DZX**	74.3 ± 13.6	74.4 ± 9.8	129.9 ± 33.6	85.2 ± 15.3
**HTN+EX+DZX**	156.06 ± 21.2	133.1 ± 29.9	182.9 ± 16.5	109.9 ± 30.2

The data are expressed as a mean ± standard deviation (SD); $n = 5$. Effects were evaluated using factorial ANOVA. When interactions were detected, Tukey post hoc paired comparisons were completed and reported such that $^*p \le 0.05$ before fatigue versus post-fatigue.
